# A Dose-Finding Trial for Hyperthermic Intraperitoneal Cisplatin in Gynecological Cancer Patients Receiving Hyperthermic Intraperitoneal Chemotherapy

**DOI:** 10.3389/fonc.2021.616264

**Published:** 2021-03-11

**Authors:** Chui-ying Chan, Hui Li, Miao-fang Wu, Chang-hao Liu, Huai-wu Lu, Zhong-qiu Lin, Jing Li

**Affiliations:** Department of Gynecologic Oncology, Sun Yat-sen Memorial Hospital, Sun Yat-sen University, Guangzhou, China

**Keywords:** Bayesian optimal interval design, dose-finding trial, cisplatin, gynecological cancer, hyperthermic intraperitoneal chemotherapy, kidney injury, maximum tolerated dose

## Abstract

**Background:** To identify the maximum tolerated dose (MTD) of hyperthermic intraperitoneal cisplatin at 43°C among gynecological cancer patients.

**Methods:** In this Phase I dose-finding trial, Bayesian optimal interval (BOIN) design was used. We sought to explore the MTD with a target dose-limiting toxicity (DLT) rate of 20%, 4 prespecified doses (70 mg/m^2^, 75 mg/m^2^, 80 mg/m^2^ and 85 mg/m^2^), and 30 patients.

**Results:** Between 2019 and 2020, 30 gynecologic cancer patients were enrolled. No patients received bevacizumab in subsequent treatment. The most common adverse events related to cisplatin were nausea and vomiting (100%), followed by tinnitus (26.7%) and kidney injury (23.3%). Of the seven patients with kidney injury, four had persistent renal impairment, and finally progressed into chronic kidney injury. DLTs were noted only in the dose level 4 group (85 mg/m^2^) and included acute kidney injury, pulmonary embolism, anemia, and neutropenia. When cisplatin was given at dose level four (85 mg/m^2^), the isotonic estimate of the DLT rate (22%) was closest to the target DLT rate of 20%. Therefore, 85 mg/m^2^ was selected as the MTD, with a 51% probability that the toxicity probability was greater than the target DLT rate.

**Conclusions:** For gynecological cancer patients who received HIPEC for peritoneal metastases, the MTD of cisplatin in HIPEC at 43°C was 85 mg/m^2^. Our findings apply to patients who do not receive bevacizumab (ChiCTR1900021555).

## Introduction

Peritoneal metastasis (PM) is a common manifestation of gynecological carcinomas and has significantly negative influence on patient prognosis (1). For PM patients, a combination of cytoreduction and hyperthermic intraperitoneal chemotherapy (HIPEC) is an important treatment option. Malignant cells can be killed by hyperthermia in the range of 41–43°C (2). Heat can also change drug pharmacokinetics and increase the cytotoxicity of certain cytotoxic drugs (1). In addition, previous clinical studies have noted improved survival outcomes among PM patients treated with HIPEC (3–5). On the basis of this evidence, many guidelines recommend HIPEC for gynecological cancer patients with PM (6–8).

Cisplatin is one of the first-line drugs used in HIPEC (6, 7). However, the optimal cisplatin dose regimen for HIPEC is still uncertain. The reported doses of cisplatin in the literature vary significantly, ranging from 15 to 150 mg/m^2^ (1, 9). The recommend dose in the National Comprehensive Cancer Network (NCCN) guidelines is 100 mg/m^2^ (6), which is based on a randomized controlled trial (RCT) in which 245 ovarian cancer patients were randomized to receive interval debulking surgery (IDS) either with or without HIPEC (4). Although the authors reported that the addition of HIPEC did not result in an increased rates of side effects, several aspects of this trial deserve attention. First, the authors did not detail cisplatin-related adverse events (AEs) completely. Second, all patients received sodium thiosulphate during the procedure of HIPEC to alleviate cisplatin-related nephrotoxicity, so the real nephrotoxicity induced by hyperthermic cisplatin remains unclear. Finally, the administration of cisplatin in the trial was according to the following schedule: 50% of the dose at start, 25% at 30 min and 25% at 60 min. In this way, the maximum dose in the abdomen was lower than 100 mg/m^2^. Collectively, it is hard to reach a definite conclusion that a dose of 100 mg/m^2^ of cisplatin is safe for HIPEC. Since most cisplatin-related AEs are dose dependent (10), the lack of a standard dose has a major impact on the safe application of HIPEC. Two dose-finding trials for cisplatin in HIPEC have been published (11, 12). However, the designs of these trials are methodologically arguable. The two trials enrolled only European patients, and many patients received bevacizumab following HIPEC. Given the influence of race differences and the nephrotoxicity of bevacizumab (13–15), the conclusions of those studies may not be generalizable to other patient groups. Additionally, the treatment temperatures of HIPEC in the published literature are different from the recommended temperature in the Chinese Anti-cancer Association (CACA) guidelines (7). Since the toxicity of some chemotherapeutic drugs is temperature dependent (16), it is necessary and important to explore the dosing regimen at a given temperature. Herein, utilizing Bayesian optimal interval (BOIN) design, we initiated a Phase I dose-finding trial to explore the maximum tolerated dose (MTD) of hyperthermic intraperitoneal cisplatin at 43°C in gynecological cancer patients.

## Materials and Methods

### Study Design and Patient Eligibility

The present study is an open-label Phase I dose-finding trial which was carried out at Sun Yat-sen Memorial Hospital. The study was conducted according to the Declaration of Helsinki. The study protocol was approved by the Institutional Review Board of Sun Yat-sen Memorial Hospital and registered at Chinese Clinical Trial Registry (ChiCTR, http://www.chictr.org.cn/abouten.aspx, ChiCTR1900021555). Registration occurred before the start of the trial and before any patients were enrolled. This article adheres to the CONSORT (Consolidated Standards of Reporting Trials) guidelines. All patients provided signed written informed consent obtained before enrollment. Patients were recruited at the Department of Gynecologic Oncology of Sun Yat-sen Memorial Hospital from January 2019 and January 2020.

The primary objective of the present study was to identify the MTD for hyperthermic intraperitoneal cisplatin at 43°C. Secondary outcomes were hyperthermic cisplatin induced serious AEs that mainly include major kidney dysfunction that required emergent dialysis and visceral perforation (11, 13, 17) and short-term survival outcomes.

Gynecological cancer patients who fulfilled the indication of HIPEC according to CACA guidelines were eligible if they met all the following criteria (7): age between 18 and 65 years; adequate renal function (blood creatinine: 58–96 μmol/L), bone marrow function (hemoglobin ≥ 110 g/L, white cell count ≥ 4.0 × 10^9^/L, neutrophil count ≥ 2.0 × 10^9^/L, platelet count ≥ 100 × 10^9^/L) and hepatic function [bilirubin 3.4–22.2 μmol/L, alanine aminotransferase (ALT) 7–40 U/L, aspartate aminotransferase (AST) 13–35 U/L, AST/ALT ≤ 1.5]. Patients were excluded if they had been treated with cisplatin for any reason within 3 weeks prior to HIPEC.

### Treatment Plan

Following cytoreduction or biopsy, four tubes were placed (two in the bilateral subdiaphragmatic space for use as inlet tubes and two in the pelvic cavity for use as an outlet tubes) which were used to administrate HIPEC (18). Due to limited HIPEC equipment, we initially had planned to administer HIPEC within 48 h of surgery, but all patients received HIPEC immediately after surgery while the trial was actually being carried out. Cardiac, renal, hepatic, and bone marrow function were re-evaluated prior to the initiation of HIPEC. A high precision hyperthermic intraperitoneal perfusion treatment system (approved by the State Food Drug Administration of China, approval No. 2009-3260924) was used. The system has a precision of ±0.10°C for temperature control and ±5% for flow control. Cisplatin was added to 3,000–5,000 mL of saline solution and administered at 43 ± 0.10°C. The HIPEC procedure consisted of a 30 min preheating period and a 60 min perfusion period. During the treatment, vital signs and urine output were monitored continually. Intravenous hydration was required for all patients. It was started 1 h before the initiation of HIPEC and maintained until 24 h after the completion of treatment. After HIPEC, follow-up visits were scheduled weekly for the first 3 weeks, then every 3 weeks for 3 months and every 3–6 months thereafter.

### Statistical Analysis

**Figure 1:**
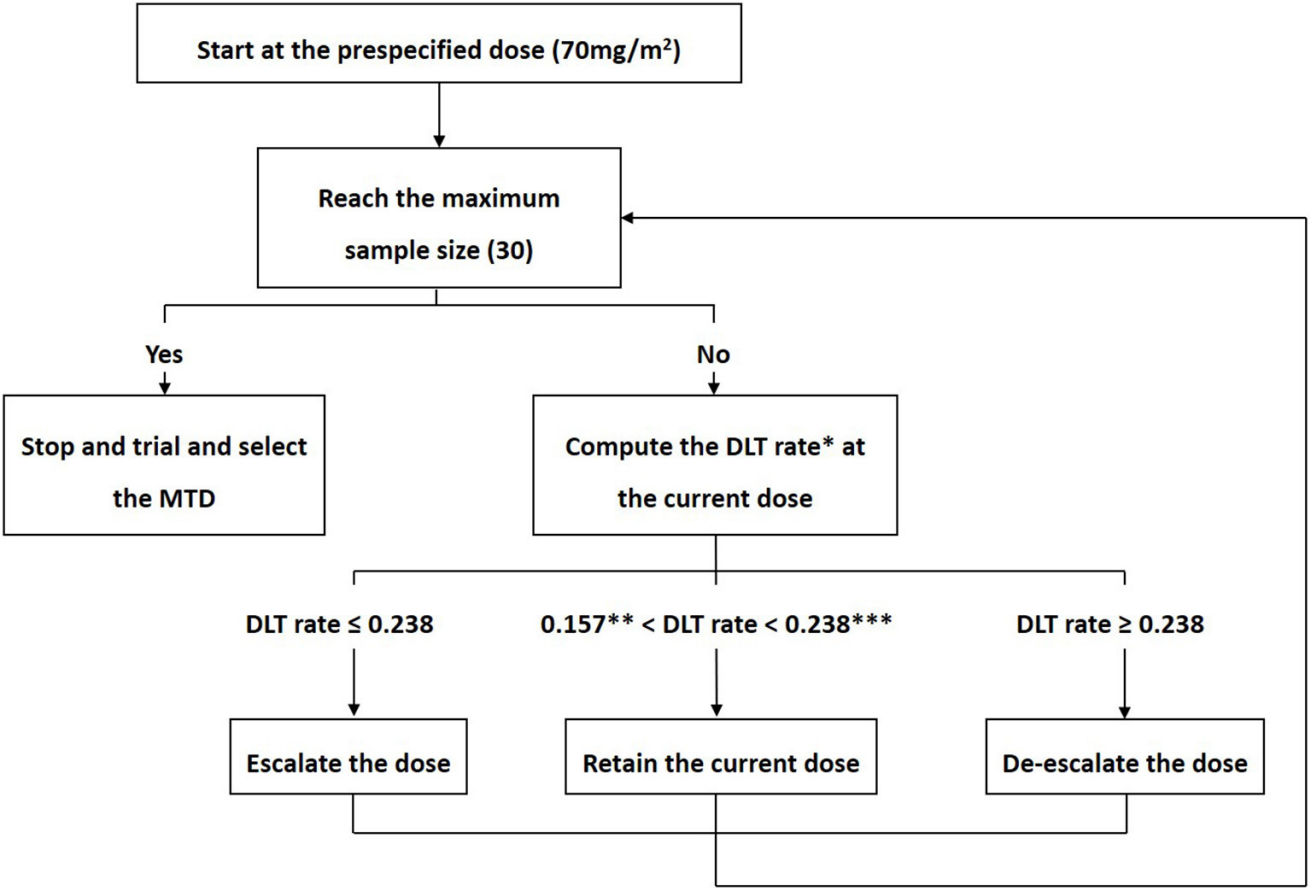
Bayesian optimal interval design. According to the Bayesian optimal interval (BOIN) design, the decision to escalate or de-escalate the dose was made by a comparison of the observed dose-limiting toxicity (DLT) rate ($\hat{p}$) at the current dose with fixed prespecified dose escalation (λ_*e*_) and de-escalation (λ_*d*_) boundaries. We sought to explore the maximum tolerated dose (MTD) with a target DLT rate of 20%, 4 prespecified doses (70, 75, 80, and 85 mg/m^2^) and 30 patients. The corresponding dose escalation and de-escalation boundaries were λ_*e*_ = 0.157 and λ_*d*_ = 0.238, respectively. The trial protocol was generally described as follows. (1) The first cohort of three patients received cisplatin at a dose of 70mg/m^2^. (2) If $\hat{p}$ ≤ λ_*e*_, the dose was escalated; If $\hat{p}$ ≥ λ_*d*_, the dose was de-escalated; If λ_*e*_<$\hat{p}$ < λ_*d*_, the current dose was retained. (3) Step 2 was repeated until the maximum sample size was reached. *DLT rate = $\frac{Total\;number\;of\;patients\;who\;experienced\;DLT\;at\;the\;current\;dose\;}{Total\;number\;of\;patients\;taking\;the\;current\;dose}$ **0.157, λc, which represents the dose escalation boundary. ***0.238, λd, which represents the dose de-escalation boundary. MTD, maximum tolerated dose; DLT, dose-limiting toxicity.

The MTD was explored with a target DLT rate of 20%, 4 prespecified doses (70, 75, 80, and 85 mg/m^2^) and 30 patients. Patients were treated in cohorts of three. A BOIN design was used to identify the MTD of escalating doses of cisplatin (19). Figure 1 shows the BOIN design and summarizes the trial protocol. The Data and Safety Monitoring Board (DSMB) reviewed all toxic effects and made decisions on dose escalation. Cisplatin-related serious AEs during HIPEC have been reported in many studies, including major kidney dysfunction that required emergent dialysis and visceral perforation (11, 13, 17). Considering these reports and patient safety, DSMB recommended the use of grade 3 AEs, rather than grade 4 AEs, to define DLT. When the trial was completed isotonic regression was performed which pooled information across doses and obtained an estimate of the MTD. DLT was defined as toxic effects that were associated with cisplatin within 3 weeks following HIPEC, which included (1) death; (2) digestive, hepatobiliary or pancreatic perforation or fissure requiring surgery; (3) hemorrhage requiring transfusion or surgery; (4) life-threatening or irreversible disability; and (5) grade ≥3 adverse events according to the National Cancer Institute Common Terminology Criteria for Adverse Event (CTC-AE) Version 4.0 classification. Kidney injury was divided into acute kidney injury (AKI), which was observed within 3 days of HIPEC, and late-onset kidney injury (LKI), which was observed within 3–21 days after HIPEC. If kidney injury lasted more than 3 weeks, it was considered chronic kidney injury (CKI). Post-HIPEC complications that were attributable to surgery were not considered DLTs.

The calculation of dose elimination boundaries, the estimation of the 95% exact confidence interval (CI) for the toxicity rate and the selection of the MTD were completed using the BOIN Design Desktop Program, which was downloaded from the MD Anderson Software Download website (https://biostatistics.mdanderson.org/SoftwareDownload), as well as an online BOIN design tool (https://ibl.mdanderson.org/BOIN/). Baseline patient characteristics were described with descriptive statistics and analyzed using Stata statistical software (version 15.0, Stata Corp LP, College Station, TX). All statistical tests were tow-sided, and differences were considered statistically significant at *P* < 0.05.

## Results

**Figure 2:**
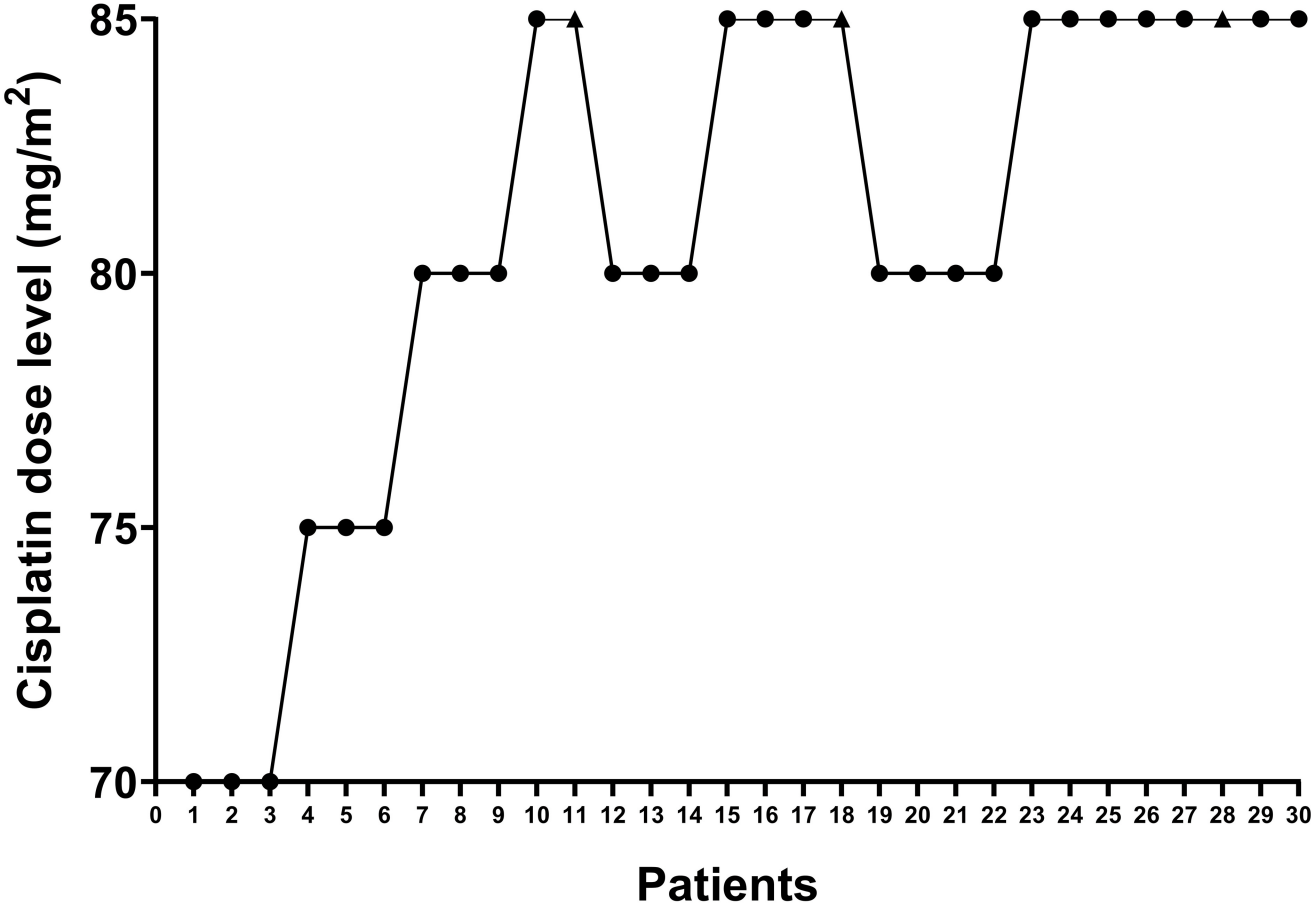
Enrollment of study patients. The *X*-axis shows individual patients who were enrolled sequentially. The *Y*-axis represents the four dose levels (70, 75, 80, and 85 mg/m^2^). The black dots indicate patients who did not develop dose-limiting toxicity, while the black triangles indicate patients who developed dose-limiting toxicity.

Between January 2019 and January 2020, 30 gynecological cancer patients were enrolled, and received HIPEC right after surgery. Figure 2 shows the enrolling procedure. The clinicopathologic and treatment characteristics of the patients are listed in Table 1. Twenty-seven (90%) patients had a pathological diagnosis of primary ovarian cancer, while 3 (10%) patients had PM from other sites. Before HIPEC, 9 (30%) patients received only biopsy because of unresectable disease, and they underwent two cycles of intravenous neoadjuvant chemotherapy followed by interval debulking surgery. All 30 patients received subsequent cycles of planned chemotherapy within 3–4 weeks of HIPEC without dose delay or dose reduction, and no patient received bevacizumab.

**Table 1:** Descriptive features of patients, tumors and surgical outcomes.

**Variable**	
Age (years), median (range)	51 (32–65)
Body surface area (m^2^), median (range)	1.55 (1.28–1.78)
BMI (kg/m^2^), median (range)	22.6 (16.4–28.4)
**Histopathology**, ***n*** **(%)**	
Ovarian cancer	27 (90.0)
Mucinous adenocarcinoma	3 (11.1)
Serous adenocarcinoma	19 (70.4)
Endometrioid adenocarcinoma	1 (3.7)
Clear cell adenocarcinoma	2 (7.4)
Granulosa cell tumor	1 (3.7)
Malignant germ cell tumors	1 (3.7)
Endometrial cancer	1 (3.3)
Cervical adenocarcinoma	1 (3.3)
Peritoneal mesothelioma	1 (3.3)
**Tumor histologic grade**, ***n*** **(%)**	
Grade 1–2	21 (70.0)
Grade 3	9 (30.0)
pre-HIPEC serum creatinine (umol/l), median (range)	64.5 (43–93)
pre-HIPEC serum albumin (g/l), median (range)	33.5 (22.2–48.6)
Peritoneal cancer index, median (range)	6 (1–30)
**Surgical procedures**, ***n*** **(%)**	
Hysterectomy	13 (43.3)
Salpingo-oophorectomy	17 (56.7)
Lymphadenectomy^*^	8 (26.7)
Small bowel resection	1 (3.3)
Hepatectomy	2 (6.7)
Omentectomy	17 (56.7)
Appendectomy	12 (40.0)
Diaphragmatic peritonectomy	3 (10.0)
Operation time (minutes), median (range)	172 (25–360)
Estimated blood loss (ml), median (range)	50 (1–700)
**Blood transfusion during surgery**, ***n*** **(%)**	
Yes	4 (13.3)
No	26 (86.7)
Completeness of cytoreduction^**^, ***n*** **(%)**	
CC-0	18 (85.7)
CC-1+	3 (14.3)
**ICU stay**, ***n*** **(%)**	
Yes	1 (3.3)
No	29 (96.7)

*BMI, body mass index; HIPEC, Hyperthermic intraperitoneal chemotherapy; ICU, intensive care unit*.

* *including pelvic and para-aortic lymphadenectomy*.

** *21 patients received debulking surgery*.

Table 2 details AEs that were noted within 3 weeks of HIPEC. The most common AEs were nausea and vomiting (100%), followed by tinnitus (26.7%) and kidney injury (23.3%). No anastomotic leakage or neutropenic fever was observed. The symptoms in patients with nausea and vomiting were relieved within 6–12 h of symptomatic treatment. Of the seven patients with kidney injury, three (42.8%) patients had AKI, and four (57.1%) had LKI; however, none of them required dialysis. For the four cases of LKI, the diagnosis was made on day 6, 20, 22, and 24 after HIPEC. Two of the three AKI patients and two of the four LKI patients had persistent kidney dysfunction and eventually progressed into CKI. All 30 patients received subsequent cycles of chemotherapy within 3–4 weeks of HIPEC.

**Table 2:** Adverse events according to dose level.

**Adverse event, *n***	**NCI-CTCAE 4.0**			
	**Grade 1**	**Grade 2**	**Grade 3**	**Grade 4**
70 mg/m^2^ (*n* = 3)				
hromboembolic events^¶^	–	1	–	–
Bloating	–	1	–	–
Nausea and vomiting	3	–	–	–
**75 mg/m**^**2**^ **(*****n*** **=** **3)**				
Late-onset kidney injury	–	1^*^	–	–
Intestinal obstruction	–	1	–	–
Tinnitus	2	–	–	–
Nausea and vomiting	3	–	–	–
**80 mg/m**^**2**^ **(*****n*** **=** **10)**				
Acute Kidney Injury	1	–	–	–
Late-onset kidney injury	2^**^	–	–	–
Neutropenia	–	1	–	–
Intestinal obstruction	1	–	–	–
Tinnitus	2	–	–	–
Nausea and vomiting	10	–	–	–
**85 mg/m**^**2**^ **(*****n*** **=** **14)**				
Neutropenia	–	–	1	–
Anemia	–	–	1	–
Acute Kidney Injury	–	1^*^	1^*^	–
Late-onset kidney injury	–	1	–	–
Thromboembolic events^¶^	–	2	1	–
Dizziness	1	–	–	–
Hypokalemia	–	2	–	–
Hypocalcemia	–	2	–	–
Hepatic function impairment	–	4	–	–
Tinnitus	4	–	–	–
Nausea and vomiting	14	–	–	–

*NCI-CTCAE, National Cancer Institute Common Terminology Criteria for Adverse Events*.

¶ *Thromboembolic events included venous thrombosis and pulmonary thrombosis*.

* *The patient progressed into chronic kidney injury*.

** *One patient progressed into chronic kidney injury*.

Among patients who received a cisplatin dose level ≤ 80 mg/m^2^, no grade 3 or higher AEs were observed. Three patients developed DLTs that included AKI (grade 3), pulmonary embolism (grade 3), anemia (grade 3) and neutropenia (grade 3). All three patients were in the 85 mg/m^2^ dose group. Based on the observed data, the isotonic estimates of toxicity probabilities of the four doses were 0.01 (exact 95% CI: 0.00–0.20), 0.01 (exact 95% CI: 0.00–0.20), 0.01 (exact 95% CI: 0.00–0.06), and 0.22 (exact 95% CI: 0.00–0.46). When cisplatin was given at dose level four (85 mg/m^2^), the isotonic estimate of the DLT rate (22%) was closest to the target DLT rate of 20%. Therefore, 85 mg/m^2^ was selected as the MTD with a 51% probability that the toxicity probability was greater than the target DLT rate.

The median follow-up time was 8.6 months (range: 3.0–14.6 months). One patient in the 70 mg/m^2^ dose group developed recurrence 10 months following HIPEC. Two patients died of disease. One patient received cisplatin at a dose of 80 mg/m^2^ and died of the disease 12 months following HIPEC. The other patient was treated with cisplatin at a dose of 85 mg/m^2^ and succumbed to her disease 6 months after HIPEC.

## Discussion

Cisplatin is the preferred agent for HIPEC because of its excellent peritoneal plasma gradient and its synergistic relationship with heat (2, 20). Two dose-finding studies have been published so far that identified the MTD for cisplatin among patients who received HIPEC (11, 12). In Zivanovic's study (12), three dose levels (60, 80, and 100 mg/m^2^) at 41–43°C were investigated, but no definitive MTD was identified. Favorable pharmacokinetic and pharmacodynamic properties of hyperthermic cisplatin were confirmed at all dose levels, especially at 100 mg/m^2^. Since DLT was observed only in the third dose level (100 mg/m^2^), the authors concluded that hyperthermic intraperitoneal cisplatin at a dose of 100 mg/m^2^ has an acceptable safety profile and can be considered the recommended Phase II dose. The other trial by Gouy et al. (11) reached a different conclusion. The authors assessed four dose levels (50, 60, 70, and 80 mg/m^2^) at 42 ± 1°C. They selected 80 mg/m^2^ as the MTD, as all DLTs were noted at this dose level. However, based on the observed DLTs and prolonged impairment of renal function, Gouy et al. (11) recommended a cisplatin dose of 70 mg/m^2^ for HIPEC. Overall, previous studies have yielded no consistent results or conclusions. Variations in ethnical restrictions, patient selection criteria, HIPEC regimens, treatment temperatures and study designs may contribute to the inconsistency.

In the present study, we assessed four cisplatin dose levels at 43°C, and identified 85 mg/m^2^ as the MTD. In general, all patients tolerated HIPEC well. No patient developed serious AEs that were reported in previous studies (11, 13). Of note, because of unresectable disease, 9 (30%) patients in the present trial received biopsy rather than debulking surgery prior to HIPEC. For them, HIPEC could be considered as part of neoadjuvant treatment. The safety and feasibility of HIPEC in the neoadjuvant setting for gynecologic cancer patients has been reported in our previous study (18). The most notable difference between the current trial and previous studies is the study design. The current trial was based on the BOIN design. Compared with the classical 3+3 design, which was utilized in Zivanovic's study (12), the BOIN design has superior flexibility (21). The latter design can target any prespecified DLT rate and make decisions regarding dose escalation and de-escalation at any time as long as the DLT rate at the current dose can be calculated. In addition, the BOIN design is more likely to correctly select the MTD and allocate more patients to the MTD group when compared with the 3+3 design (21). Yuan's numerical study also showed that the BOIN design generally outperforms the 3+3 design (21). With regard to the continual reassessment method (CRM), which was utilized in Gouy's trial, it has a comparable performance with the BOIN design (19). However, the CRM is statistically and computationally complex and lacks transparency hindering its use in practice (19, 21).

Cisplatin-induced kidney injury in HIPEC has been a focus of research. Major kidney injury is noted in 1.3–5.9% of patients and is characterized by notable ethnical differences (22–24). Sin et al. (13) conducted a retrospective study in Chinese patients who received cisplatin for HIPEC. For patients who developed grade 3 or higher kidney injury, they developed raised creatinine within 2 days of HIPEC, and the maximum creatinine elevation could be observed within 6–19 days of HIPEC. Similar findings were reported in Kusamura' paper (23). Based on the evidence, we classified kidney injury following HIPEC according to the timing of its occurrence. The incidence of kidney injury in the current study was 23.3%, which is consistent with previous reports (11, 13, 25). However, none of the cases of kidney injury needed to receive dialysis, which is different from the results of other studies (11, 13). All of our patients received continuous intravenous hydration which may help ameliorate the severity of renal damage (26, 27). On the other hand, of the seven cases of kidney injury, four had persistent renal impairment and finally progressed into CKI. This result and similar findings from other studies imply that HIPEC recipients who develop cisplatin-induced renal impairment may be at high risk for CKI (11, 13). Although CKI has a significant negative impact on patient quality of life and limits treatment options for cancer patients (28, 29), no effective preventive measure is available, which presents a considerable challenge in clinical practice.

The feasibility of combination of HIPEC and bevacizumab has been validated in ovarian cancer patients (30). However, it should be noted that no patient in our cohort received bevacizumab following HIPEC. Bevacizumab is also nephrotoxic (29). In a previous study, increases in serum creatinine levels, which are the most common consequence of cisplatin-induced renal injury, were observed among patients who received bevacizumab (31, 32). Given that heat can enhance the nephrotoxicity of cisplatin (2, 33), we believe cisplatin should be given with greater caution in HIPEC if subsequent bevacizumab is planned, and it may be appropriate to decrease the dose. Accordingly, we believe that the MTD in the present study should not be extrapolated to patients who will receive bevacizumab following HIPEC and vice versa.

The treatment effect of HIPEC comes not only from the chemotherapeutic drugs but also from the cytotoxicity of hyperthermia (34, 35). In our trial, the HIPEC was administered at 43°C, which is in accordance with the CACA guidelines (7). The safety and effectiveness of HIPEC at 43°C have been validated in previous clinical studies (18, 36, 37). Since the cytotoxicity of hyperthermia is temperature dependent and has a strong influence on the safe application of HIPEC (34, 35), it is necessary to investigate MTDs for chemotherapeutic drugs in HIPEC at a given temperature.

The sample size and the design of the present trial limited our ability to draw reliable conclusions regarding the efficacy of HIPEC. Second, 30% of the current cohort received only biopsy because of unresectable disease. The amount of residual disease can influence the penetration ability of cisplatin, and blood loss, which also impacts the risk of HIPEC-related renal dysfunction, could be different between patients receiving biopsy and patients receiving debulking surgery (38). Since we did not conduct pharmacokinetics and pharmacodynamics assessment or subgroup analysis, it is unclear whether the MTDs of hyperthermic cisplatin would be different between the two cohorts. Third, given the heterogeneity of our cohort, it is impossible to identify the type of patient who can benefit the most from HIPEC. Another limitation is the short follow-up duration. Therefore, the long-term adverse reactions that are associated with using cisplatin in HIPEC cannot be evaluated. Additionally, laparoscopic HIPEC for low-volume peritoneal metastasis has been reported in previous studies (39). An elevated intraabdominal pressure can be induced when laparoscopic technique is used, which could improve tissue penetration and thus enhance the cytotoxicity of cisplatin. Therefore, a more specialized dose regimen may be needed in this setting ADDIN EN.CITE (39).

In conclusion, we utilized a BOIN design in this Phase I dose-finding trial and established a dose of 85 mg/m^2^ as the MTD for hyperthermic cisplatin at 43°C. Given the characteristics of our patients, our findings are applicable to patients who do not receive bevacizumab. Further Phase II trials are warranted to validate our conclusion.

## Data Availability Statement

The raw data supporting the conclusions of this article will be made available by the authors, without undue reservation.

## Ethics Statement

The studies involving human participants were reviewed and approved by Institutional Review Board of Sun Yat-sen Memorial Hospital. The patients/participants provided their written informed consent to participate in this study.

## Author Contributions

C-yC and HL: data curation, methodology, formal analysis, methodology, writing – original draft, and project administration. M-fW and C-hL: data curation. JL: conceptualization, methodology, supervision, resources, and writing – review and editing. Z-qL: conceptualization, supervision, resources, and writing-review and editing. All authors contributed to the article and approved the submitted version.

## Conflict of Interest

The authors declare that the research was conducted in the absence of any commercial or financial relationships that could be construed as a potential conflict of interest.
